# Feasibility of mass cytometry proteomic characterisation of circulating tumour cells in head and neck squamous cell carcinoma for deep phenotyping

**DOI:** 10.1038/s41416-023-02428-2

**Published:** 2023-09-21

**Authors:** Karl Payne, Jill Brooks, Nikolaos Batis, Naeem Khan, Mohammed El-Asrag, Paul Nankivell, Hisham Mehanna, Graham Taylor

**Affiliations:** 1 Institute of Head and Neck Studies and Education, Institute of Cancer and Genomic Sciences, University of Birmingham, Birmingham, UK; 2 School of Biomedical Sciences, Institute of Clinical Sciences, College of Medical and Dental Sciences, University of Birmingham, Birmingham, UK; 3 Clinical Immunology Service, Institute of Immunology and Immunotherapy, University of Birmingham, Birmingham, UK; 4 Institute of Cancer and Genomic Sciences, University of Birmingham, Birmingham, UK; 5 Institute of Immunology and Immunotherapy, University of Birmingham, Birmingham, UK

**Keywords:** Head and neck cancer, Predictive markers

## Abstract

**Background:**

Circulating tumour cells (CTCs) are a potential cancer biomarker, but current methods of CTC analysis at single-cell resolution are limited. Here, we describe high-dimensional single-cell mass cytometry proteomic analysis of CTCs in HNSCC.

**Methods:**

Parsortix microfluidic-enriched CTCs from 14 treatment-naïve HNSCC patients were analysed by mass cytometry analysis using 41 antibodies. Immune cell lineage, epithelial-mesenchymal transition (EMT), stemness, proliferation and immune checkpoint expression was assessed alongside phosphorylation status of multiple signalling proteins. Patient-matched tumour gene expression and CTC EMT profiles were compared. Standard bulk CTC RNAseq was performed as a baseline comparator to assess mass cytometry data.

**Results:**

CTCs were detected in 13/14 patients with CTC counts of 2–24 CTCs/ml blood. Unsupervised clustering separated CTCs into epithelial, early EMT and advanced EMT groups that differed in signalling pathway activation state. Patient-specific CTC cluster patterns separated into immune checkpoint low and high groups. Patient tumour and CTC EMT profiles differed. Mass cytometry outperformed bulk RNAseq to detect CTCs and characterise cell phenotype.

**Discussion:**

We demonstrate mass cytometry allows high-plex proteomic characterisation of CTCs at single-cell resolution and identify common CTC sub-groups with potential for novel biomarker development and immune checkpoint inhibitor treatment stratification.

## Introduction

Liquid biopsy of blood offers a simple, non-invasive method to risk stratify patients and guide cancer therapy, particularly for cases of cancer recurrence or metastasis when tumour sampling is challenging or not possible [1]. Circulating tumour cells (CTCs) present in the blood of cancer patients are a potential rich source of multi-omic biomarkers [2], but the optimal methods for blood collection and storage, CTC enrichment and isolation, and subsequent downstream characterisation are still unclear. Therefore, standardised protocols to generate comparable datasets between laboratory or hospital sites are urgently required.

Flow cytometry is a widely used technique for studying cells in liquid suspensions. Attempts to use flow cytometry to identify CTCs have been limited by the use of fluorophore-labelled antibodies to phenotype the cells, as spectral overlap between different fluorophores limits the number of markers that can be simultaneously measured per cell. Consequently, CTC analysis has mostly used genomic and transcriptomic methods to study CTCs. The majority of these initial CTC sequencing studies have used bulk sequencing, analysing mixtures of multiple enriched CTCs together with associated contaminating leucocytes from each patient. While this approach allows the assessment of multiple genes or mutations from an entire CTC population, it lacks the depth of information afforded by single-cell analysis to identify CTC phenotypic heterogeneity and define novel, and potentially druggable, CTC sub-groups. Although several reports have presented single-CTC sequencing data [3], this approach is highly technically challenging and costly, limiting its ability to be upscaled to trials on large patient cohorts and translated to clinical practice. Furthermore, assessing the transcriptome is not a direct or reliable measure of the functional proteome contributing to a cellular phenotype [4, 5]. Notably, a key feature which transcriptomic analysis cannot assess is post-translational modifications (PTM) such as protein phosphorylation. PTMs dramatically increase proteomic diversity, regulating the activity, localisation and interactions of cellular proteins and are a fundamental mechanism through which cells modulate their state and function [6].

In head and neck cancer several large cohort studies have recently demonstrated that the presence of CTCs is a prognostic biomarker of survival outcomes [1, 7]. Moreover, dynamic changes in CTC count and changes in CTC phenotype appear to be predictive of response to treatment [8–10]. However, consistent with the wider field of CTC research, analysis of CTC phenotype in head and neck cancer has mostly been limited to gene expression profiling of crude, bulk CTC preparations or low-plex proteomics. Improved prediction and treatment stratification is likely to depend upon the ability to obtain single-cell high-plex characterisation of CTCs.

A critical, but often overlooked, component of any CTC enrichment protocol using non-fixed samples is the time to processing and the impact of the enrichment process on CTCs. Both of these factors have been shown to alter cell viability and gene/protein expression [11–14]. For a CTC-based liquid biopsy to be successful in prospective multi-centre clinical trials, and ultimately translated to clinical practice, the assay needs to be reproducible while also accommodating variable time intervals between sample collection and sample processing. Previously, we have validated the ability of Transfix blood collection tubes, which are sold pre-loaded with fixative, to immediately preserve the phenotype of CTCs at the point of sample collection. Cell phenotype was stabilised for up to 72 h post-collection, and CTCs could be isolated at any point during this time period using the Parsortix microfluidic platform [7, 14]. Here, we develop this protocol further: combining Transfix blood collection and Parsortix CTC enrichment with mass cytometry. Our primary aim was to demonstrate feasibility that mass cytometry, performed on a fixed blood sample, would allow high-plex characterisation of CTC protein expression, including phosphorylated signalling proteins, and thus identify potentially novel CTC sub-groups with druggable potential.

## Method

### Study cohorts, sample collection and sample processing

**Fig. 1:**
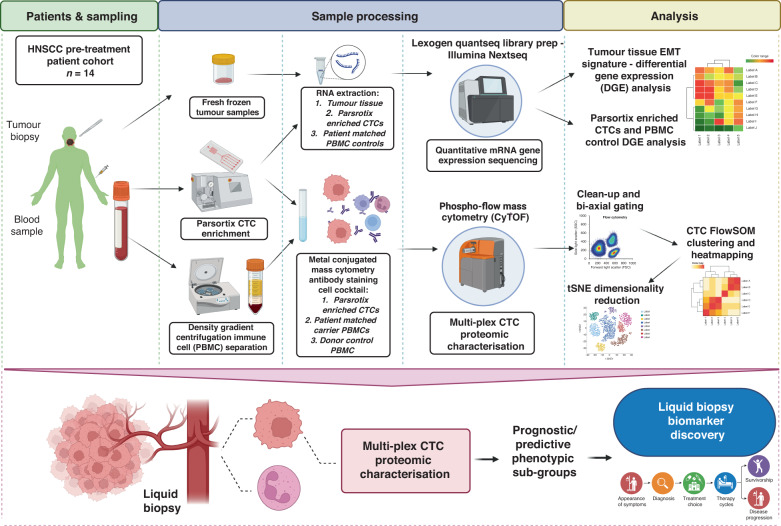
Project structure. Diagrammatic representation of project design, sample processing and data analysis strategy – including areas of future research and clinical translation (HNSCC head and neck squamous cell carcinoma, CTC circulating tumour cell, PBMC peripheral blood mononuclear cell, CyTOF cytometry by time of flight, tSNE t-distributed stochastic neighbour embedding).

Project design is shown in Fig. 1. Patients were recruited through the ethically approved Accelerated2 sample collection platform (REC ref:16/NW/0265) and provided written informed consent. Blood samples were obtained from 14 treatment naïve patients with biopsy proven HNSCC. Venous blood was sampled directly into a 9 ml Transfix blood collection tube (BCT) and a lithium heparin BCT to provide autologous peripheral blood mononuclear cells (PBMCs) for use as carrier cells during CTC antibody staining post-enrichment. Blood samples from six healthy donors were collected and processed using the same CTC isolation protocol used for patient samples to establish a baseline of CTC detection in people without cancer. PBMCs were isolated by Lymphoprep density-gradient centrifugation.

### Preparation of autologous carrier cells

To produce autologous carrier cells, 900,000 PBMCs from the patient’s heparin BCT were incubated in Transfix BCT preservative for 24 h at 4 °C to replicate the fixation conditions of the blood sampled directly into Transfix tubes (to allow downstream analysis). The PBMCs were then barcoded by staining with a mixture of cadmium-106 labelled beta-2-microglobuln and ATPase (CD298)-specific antibodies. These barcoded PBMCs were added to the patient’s Parsortix-enriched CTCs to act as carrier cells, minimising cell loss during antibody staining.

### Parsortix CTC enrichment from transfix blood samples

Transfix blood samples were stored at 4 °C and processed within 48 h of collection using the Parsortix device, following a previously optimised protocol [14]. In brief, the Transfix BCT is loaded onto the machine and run using protocol *PX2_*S99F which pressurises the whole blood at 99 bar through a tiered microfluidic cassette, designed to capture cells based upon size (larger than 6.5 μm diameter) and compressibility/deformability characteristics.

### Positive control PBMCs for phospho-flow analysis

To serve as a positive control for phospho-protein staining, PBMCs isolated from a single healthy donor were stimulated with cell activation cocktail (PMA/Ionomycin, Biolegend) for 15 min to upregulate phosphoprotein signalling pathways. These cells were then labelled with osmium tetroxide and cryopreserved in aliquots of 100,000 cells. Phosphoprotein control cells were thawed, washed twice and then immediately added to the patient’s CTC and carrier cells immediately before the first paraformaldehyde (PFA) fixation step of the phosphoprotein staining procedure. PBMCs from the same batch of positive control cells were used as a positive control for all patient samples.

### Mass cytometry antibody panel

A 41-marker antibody panel was designed and optimised for staining of Parsortix enriched Transfix fixed cells (Table 1, supplementary table 1) using a mock CTC model of HNSCC cell lines spiked into healthy donor blood (the HPV-negative FaDu (ATCC HTB-43) and CAL27 (ACC-446; DSMZ) cell lines kindly gifted by Prof. J. de Winter, Amsterdam). The panel was designed to allow immune cell-lineage discrimination and to detect the following: epithelial-mesenchymal transition (EMT), cell stemness potential, cell proliferation state, immune checkpoint expression, immune evasion potential and phosphorylated signalling proteins relevant to HNSCC.

**Table 1:** Antibody panel for mass cytometry analysis of CTC and PBMCs.

Cell lineage markers		Stemness/Proliferation	
EpCAM	Epithelial	CD133	Stemness
Pan-cytokeratin	Epithelial	CD24	Stemness/B-cell
E-cadherin	Epithelial	CD44	Stem/proliferation
EGFR	Epithelial	Ki67	Proliferation
CD3	T-cell	Immune evasion/suppression	
CD4	CD4+ T-cell	CD47	Immune evasion
CD8	CD8+ T-cell	PD-1	Immune response
CD25	T-regs	PD-L1	Immune suppression
CD14	Monocytes	PD-L2	Immune suppression
CD86	M1 Macrophage	CTLA4	Immune suppression
CD163	M2 Macrophage	CD39	Immune suppression
CD19	B-cells	CD73	Immune suppression
CD56	NK cells	Phosphorylated signalling proteins	
CD66b	Granulocytes	pSTAT1	Immune surveillance
HLA-ABC	MHC class I	pSTAT3	Proliferation
HLA-DR	MHC class II	pSTAT5	Cell survival
CD45	Pan leucocyte	pPARP	DNA damage repair
CD31	Endothelial cells	pAKT	Proliferation
Epithelial-mesenchymal transition		pERK	Proliferation
Vimentin	Mesenchymal	pCREB	Proliferation/survival
Snail1	EMT	p38	Proliferation/survival
Twist	EMT	*B2M* *+* *ATPase*	*PBMC carrier barcoding*

Chosen markers are shown in sub-groups depicting each set with the purpose of a) primarily identifying CTCs and immune cells and then b) phenotype these cells and characterise signalling pathways.

### Antibody staining procedure and mass cytometry analysis

For each patient, the sample to be stained with antibodies comprised of the following: i. the Parsortix enriched cells (number varying for each patient but always between 20,000–50,000 cells per patient); ii. autologous PBMC carrier cells (900,000 cells); and iii. osmium-labelled activated control cells (100,000 cells). Samples were frozen in media containing 10% DMSO and 90% FCS and stored at −80 °C. Cells were analysed in batches on a Fluidigm Helios mass cytometer. Cells were removed from −80 °C storage, thawed, washed twice with deionized distilled water, and filtered through a 70 μm filter. Four element calibration beads (Fluidigm) were added immediately prior to acquisition to allow data normalisation using the Helios acquisition software. During acquisition the event acquisition rate was maintained below 500 events/second.

### Mass cytometry data analysis and CTC identification

Normalised mass cytometer FCS (flow cytometry standard) files were analysed using the online OMIQ platform. ArcSinh transformed files underwent clean-up gating as described by Bagwell et al. [15] using Gaussian discrimination parameters. Next, B2M/ATPase barcoded PBMC carrier cells, Osmium-labelled phosphoprotein control cells and Parsortix enriched cells were separated into distinct populations by top-level gating using biaxial plots. CTCs were identified within the Parsortix enriched population as cells that have low expression of CD45, and immune lineage markers (CD3, CD4, CD8, CD19, CD14, CD56, CD66b) and intermediate to high expression of the epithelial marker pan-cytokeratin. To allow inter-patient CTC comparison and higher clustering, a single dataset was then generated by concatenating CTC events from all CTC positive patients. Unsupervised clustering analysis of the concatenated CTC dataset was performed using FlowSOM (elbow meta-clustering). Median metal intensity of CTC target markers in each FlowSOM derived cluster was assessed by hierarchical clustering to identify parent groups. Basic comparison of single marker expression between clustered CTC sub-groups was performed using the Mann–Whitney U test on ArcSinh transformed median metal signal intensity values.

### Tumour tissue gene expression profiling

Snap frozen tumour samples were available for 10 of the 14 patients who underwent whole blood CTC enrichment and analysis. RNA was extracted from tumour tissue samples using the Qiagen RNeasy mini kit. Gene expression profiling was performed using the Lexogen Quantseq 3’mRNA-Seq FWD Library preparation kit analysed using an Illumina NextSeq 500 sequencer. Sequencing reads were trimmed to remove adaptor contamination, polyA read through, and low-quality tails using bbduk utility according to the manufacturer’s guidelines. The trimmed reads were mapped to the GRCh38 (hg38) human genome and reads mapping to genes were counted using STAR aligner (v2.5.2b) [16]. Normalisation of read counts and differential gene expression analysis was performed with the DESeq2 R Bioconductor package [17]. Hierarchical clustering of samples was performed using pheatmap in R using euclidean distance. Samples were clustered based upon the expression of the HSNCC EMT signature validated by Jung et al. [18].

## Results

### Blood samples from HNSCC patients contain high frequencies of CTCs

To establish a threshold of CTC-like cells in healthy donors, we first tested blood from healthy donors using our previously established CTC isolation and identification protocol [19]. Cells matching the criteria of CTCs were detected in blood samples from two of the six healthy donors tested, but at a very low frequency (2 and 3 cells/ml). We then applied the same protocol to our HNSCC 14 patient cohort. Most patients were male (*n* = 10/14), had advance stage III-IV disease (*n* = 12/14) and an oral sub-site predominance (*n* = 13/14) (Table 2). We detected CTCs in 13/14 (93%) patients, at a quantity of 18-218 CTCs per sample (2–24 CTCs/ml, Table 2). One patient with stage 1 (T1N0) disease had a total of 4 CTCs; as this was similar in frequency to the healthy donor control threshold we scored this patient as ‘CTC negative’. There was no positive correlation between total CTC count, T-stage, positive nodal metastasis or overall stage of disease (simple logistic regression, *p* < 0.05).

**Table 2:** Study cohort.

Patient number	Age	Gender	T	N	M	Stage	Tumour site	Treatment	CTC count
**1**	77	M	4a	1	0	IV	Oral	S + CRT	97
**2**	65	M	4a	0	0	IV	Larynx	S	40
**3**	73	M	3	2b	0	IV	Oral	S + RT	54
**4**	75	M	3	2b	0	IV	Oral	S + RT	98
**5**	53	M	3	0	0	III	Oral	S + CRT	58
**6**	63	M	4a	0	0	IV	Oral	S + RT	167
**7**	43	F	4a	2b	0	IV	Oral	S + CRT	86
**8**	66	F	4a	2b	0	IV	Oral	S + RT	18
**9**	57	M	3	0	0	III	Oral	S	39
**10**	52	F	2	0	0	II	Oral	S	25
**11**	73	M	4a	0	0	IV	Oral	S + RT	183
**12**	76	F	1	0	0	I	Oral	S	0
**13**	61	M	4a	0	0	IV	Oral	S + RT	61
**14**	62	M	4a	2b	0	IV	Oral	S + RT	218

Clinical-pathological data, treatment received and total CTC count (obtained from 9 ml blood) for HNSCC patients in our study cohort (S = surgery, CRT = chemoradiotherapy, RT = radiotherapy).

### Mass cytometry and unsupervised clustering identifies epithelial and EMT sub-groups of CTCs

**Fig. 2:**
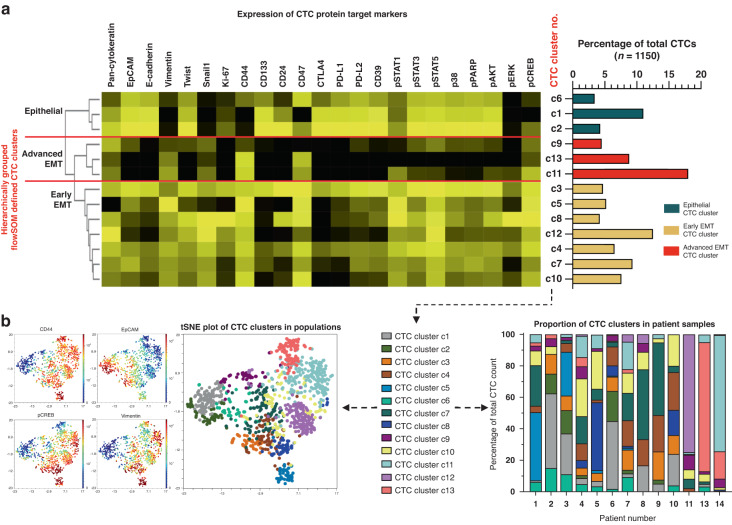
Unsupervised clustering of CTCs identifies sub-groups based upon EMT marker expression. **a** Heatmap of results from unsupervised FlowSOM clustering (undefined k value, elbow metaclustering) of concatenated mass cytometry CTC data (1150 CTCs from *n* = 13 patients) identified 13 clusters, shown as individual rows (yellow = high signal, black = low signal). Hierarchical clustering of these FlowSOM clusters identified three distinct CTC groups; based on CTC marker expression there were classified as ‘epithelial’, ‘early EMT’ and ‘advanced EMT’. Frequency of CTCs within each cluster shown as bar chart on right. **b** tSNE dimensionality reduction plots of all CTCs coloured by expression of CD44, EpCAM, phospho-CREB or vimentin or the cluster assigned to each cell (left panels) and the proportion of each patient’s CTCs assigned to each CTC cluster (right panel).

**Fig. 3:**
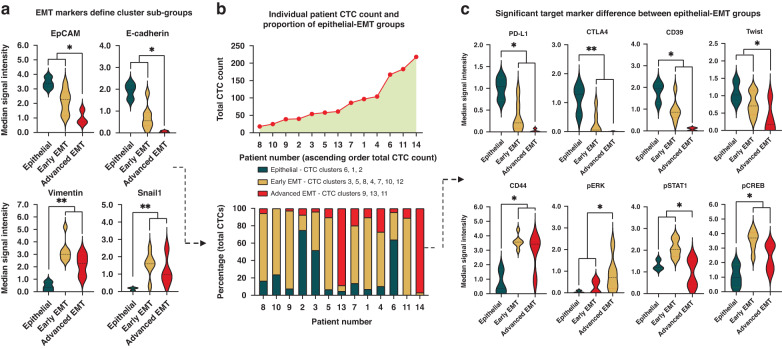
Significant differences in phenotype and cell signalling pathway activity between CTC EMT subgroups. **a** Differences in EMT markers between the epithelial, early EMT and advanced EMT subgroups of CTCs. **b** Total CTC count and CTC epithelial-EMT subgroup composition for each patient. **c** Significant differences in levels of phenotypic markers and intracellular signalling activation between epithelial, early EMT and late EMT CTCs. *p < 0.05, **p < 0.01 calculated by unpaired Mann–Whitney U test, *n* = 1150 CTCs from 13 HNSCC patients (green = epithelial, orange = early EMT and red = advanced EMT CTCs. Y-axis in panel **a** and **c** represents ArcSinh transformed median signal intensity (corresponding to CTC cluster marker expression).

We identified a total of 1150 CTCs in the blood of the 13 CTC-positive HNSCC patients. Unsupervised FlowSOM clustering of the mass cytometry data from all CTCs generated 13 clusters. As per our CTC identification strategy all CTCs had intermediate/high pan-cytokeratin expression as a baseline. Subsequent hierarchical clustering divided the 13 clusters into three CTC sub-groups, largely on the basis of differences in epithelial-EMT marker expression (Figs. 2 and 3). We labelled cells in the first group as ‘epithelial CTCs’ due to high expression of the epithelial markers pan-cytokeratin, EpCAM and E-cadherin but low expression of vimentin, a marker of epithelial-mesenchymal transition (EMT). We labelled cells in the other two groups as ‘EMT’, based on their expression of vimentin, and divided them into ‘early-EMT CTCs’ or ‘advanced-EMT CTCs’ based on EpCAM and E-cadherin expression at moderate or low levels respectively.

The early EMT and advanced EMT CTCs were most abundant, accounting for 50% (575/1150) and 31.3% (360/1150) of cells respectively. The remaining 18.7% (215/1150) of cells were epithelial CTCs. Each patient’s CTC composition was highly variable (Fig. 3). All patients apart from two (patients 10 and 11) possessed CTCs belonging to all three CTC groups. Comparison of CTC sub-group composition (i.e. epithelial, early- or advanced EMT) to T-stage and overall stage was not possible in our cohort skewed towards advanced stage disease. The presence of nodal metastasis did not correlate with the presence of EMT CTCs (t-test, *p* = 0.265).

### Phenotypic expression patterns between epithelial and EMT sub-groups of CTCs

Examining the expression of phenotypic markers and phosphorylation state of cell signalling proteins in CTCs, we identified additional differences between the epithelial, early EMT and advanced EMT groups of cells (Fig. 3a, c). As expected, levels of Snail1, a marker of EMT, were significantly higher in early and advanced EMT CTCs compared to epithelial CTCs. In contrast, expression of another EMT marker, Twist, was significantly higher in early EMT CTCs compared to advanced CTCs. Regarding other phenotypic markers, compared to epithelial CTCs the early and advanced EMT CTCs had significantly higher expression of CD44, a marker linked to stemness and invasion [20, 21].

Overall, expression of immune checkpoints, such as PD-L1, CTLA4 and CD39 were significantly reduced in early and advanced EMT groups when compared to epithelial groups. However, a select few individual EMT CTC sub-groups demonstrated increased immune checkpoint expression, which appeared to correlate with other cell phenotype expression patterns – such as increased Ki-67, CD44 and CD47 expression (for example CTC cluster c3 or c4, Fig. 2). In general, expression of stemness markers CD133 and CD24 was low in EMT sub-groups compared to epithelial sub-groups. In addition, CD47 appeared to be inversely related to stemness marker expression, with stemness high epithelial sub-groups being CD47 low.

When examining the proliferation marker Ki-67, no significant patterns emerged between epithelial and EMT groups. As a general observation, Ki-67 expression was low in advanced EMT sub-groups, intermediate in epithelial sub-groups, and increased in a select few early EMT sub-groups. Of note, within these Ki-67 high, early EMT sub-groups, cells with both immune checkpoint low and high expression were observed.

### Intracellular signalling pathway activity varies across CTC groups

Our mass cytometry panel also revealed that differences in phosphorylated signalling protein levels existed between the three CTC subgroups, indicating they also differed in cell signalling pathway activity. As a general observation, levels of phosphorylated signalling proteins, including pSTAT3 and pSTAT5, pPARP, and pAKT were higher in epithelial CTCs compared to advanced EMT groups, with a mixed picture in early EMT (Fig. 2a). pSTAT1 was significantly increased in epithelial and early EMT groups compared to advanced EMT groups (*p* < 0.05, Fig. 3c). A notable pattern was pCREB, which was significantly higher in both early and advanced mesenchymal transitioning CTCs when compared to epithelial sub-groups (*p* < 0.05, Fig. 3c). In addition, pERK was highly expressed in EMT CTCs, but with a significant increase in early EMT groups compared to epithelial and advanced EMT groups (*p* < 0.05, Fig. 3c).

### Validation of mass cytometry characterisation against conventional bulk CTC gene expression profiling

**Fig. 4:**
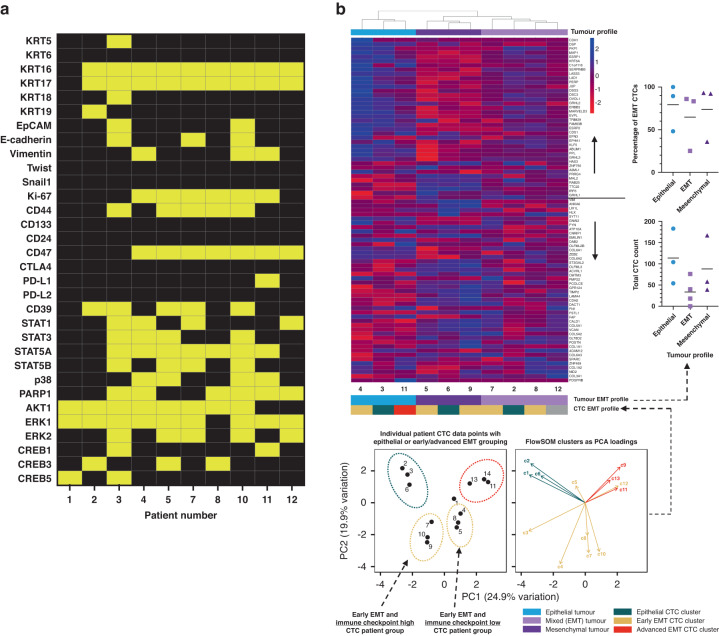
Mass cytometry outperforms CTC bulk RNAseq and tumour epithelial-mesenchymal profile does not correlate with patient CTC EMT status. **a** Differential gene expression (DGE) analysis comparing bulk RNA extracted from Parsortix enriched blood samples to patient matched PBMC negative controls, identifies genes with significantly elevated transcript counts in patient CTC samples. Results of CTC target marker gene expression corresponding to the mass cytometry protein panel are shown listed as columns (yellow = significant DGE, black = no significant DGE). **b** Heatmap of gene expression of the HNSCC EMT signature for tumour samples from our patient cohort (left), with mesenchymal genes below and epithelial genes above. Samples are hierarchically clustered to reveal epithelial, mesenchymal and mixed i.e. EMT tumour groups. Graphical results of CTC count and EMT status compared to tumour profile are shown (right), neither of which was statistically related. PCA analysis of FlowSOM derived CTC cluster composition for each patient separates patients into clear epithelial-EMT groups of based upon cluster loadings (bottom). Note early EMT patients separated by levels of immune checkpoint marker expression.

Bulk gene expression profiling (GEP) of CTCs enriched from blood is widely used to study CTC biology. To validate our protocol against this current standard we performed bulk CTC GEP of the Parsortix enriched cell population from 10 patients within our study (examining blood collected at the same timepoint as mass cytometry characterisation). Differential expression of genes (compared to patient-matched PBMC control values) corresponding to CTC markers in the mass cytometry panel were plotted for each enriched sample (Fig. 4a). Expression of epithelial transcripts, corresponding to the presence of CTCs, was observed in 9/10 samples. This CTC positivity correlated with mass cytometry data in 8 out of 10 samples. EMT CTCs, via the expression of Vimentin, were identified in 3 samples. Thus, we demonstrated that gene expression profiling failed to identify the presence of low frequency EMT CTCs in 5 patients when compared to mass cytometry data. Gene expression patterns (elevated or absent) of the proliferation marker Ki-67 correlated with the presence of proliferative CTC sub-groups (FlowSOM clusters c3 and c8) in mass cytometry data in 6 out of 10 samples. Patterns of CD44 and CD47 co-expression observed in mass cytometry data were maintained in gene expression profiling. The expression of stemness and immune checkpoint markers was notably absent in bulk CTC GEP, when compared to positive findings in mass cytometry data. Signalling protein gene expression was variable, with patterns observed in mass cytometry not replicated. However, samples with elevated Ki-67, CD44 and CD47 gene expression demonstrated elevated signalling protein gene expression, particularly the STAT, p38 and PARP pathways – similar in part to the pattern of ‘cell activation’ seen in select CTC clusters in mass cytometry analysis.

### Correlating tumour mesenchymal profile and CTC sub-group status

As an exploratory analysis, we investigated whether tumour mesenchymal profile correlated with CTC expression patterns - this was prompted by a lack of association between tumour and CTC expression profiles observed in previous studies [19, 22]. Tumour mRNA quantitative gene expression profiling (GEP) was carried out on core tumour samples from 10 patients in our cohort. We clustered tumour samples based upon a previously published EMT gene signature in HNSCC [18]. Based on the expression of epithelial or mesenchymal genes this produced 3 cluster sub-groups: epithelial, mesenchymal and mixed epithelial and mesenchymal gene expression i.e. an EMT profile - comprising of 3, 3 and 4 tumours respectively (Fig. 4b). Tumour mesenchymal profile did not correlate with total CTC count (ANOVA, *p* = 0.203). When investigating the EMT make-up of CTCs in patent samples compared to their tissue profile, neither the total proportion of EMT CTCs (ANOVA, *p* = 0.516) nor the proportion of early or advanced EMT CTCs (ANOVA, *p* = 0.312) correlated with tumour mesenchymal status (Fig. 4b). Furthermore, the presence of proliferative CTCs (FlowSOM clusters c3 and c8) did not appear to positively correlate with a mesenchymal tumour profile.

We used Principal Component Analysis (PCA) analysis to correlate individual patient CTC epithelial-EMT status to corresponding tumour profile. The composition of the 13 FlowSOM derived clusters in each patients CTC cohort were used as discriminating variables. Of interest, patients separated into four well-defined groups, which upon analysis of cluster loadings were observed to be epithelial, advanced ETM and two early EMT patient groups (Fig. 4b). Thus, identifying CTC heterogeneity at both the intra-patient and inter-patient level. Of note, patients in early EMT groups were separated primarily based upon clusters with high or low immune checkpoint expression. The overall individual patient epithelial-EMT CTC status, derived from the above PCA analysis, did not correlate with tumour mesenchymal profile (Fig. 4b).

## Discussion

We provide proof-of-concept that CTCs can be enriched from Transfix-collected blood samples, and analysed by mass cytometry. This method allowed us to interrogate two key CTC parameters: their phenotype, and their cell signalling pathway status. Critically, this is achieved from blood that is fixed immediately upon sampling from the patient, preventing inter-sample heterogeneity being generated during transportation to the analytical laboratory and subsequent processing related artefact. By allowing high-dimensional single-cell CTC data to be obtained from patients recruited across multiple research sites, we believe our method will accelerate clinical study recruitment and enable robust analysis of CTCs in rare cancers.

Undoubtedly the depth of biological insight derived from single-CTC sequencing methods is critically important in our understanding of the molecular biology of these metastatic cells. However, several aforementioned reasons make this approach challenging to apply to larger patient cohorts. An alternative approach taken by several groups has been to analyse CTCs proteomically. Feasibility studies describing the use of single-CTC western blotting [23] and mass spectrometry proteomics to characterise CTCs have been reported [24–26]. There are advantages and disadvantages to all these methods, not least the fact that they are complex and expensive [27]. An alternative strategy is to use well-characterised antibodies to detect key proteins of interest in CTCs, increasing plexity by labelling these antibodies with different metal isotopes, which can be readily differentiated by a mass cytometer, rather than with fluorophores that spectrally overlap. Mass cytometry has previously been used to characterise CTCs, but this required the cell sample to be immobilised onto glass slides and visualised by imaging mass cytometry [28]. Solid-phase imaging mass cytometry is an inherently slow way of analysing cells; consequently this study examined few CTCs from a single prostate cancer patient [28]. Our approach, analysing CTCs in a fluid phase using mass cytometry, has much greater cell throughput; consequently we were able to characterise a much larger number of CTCs and patients, allowing us to begin to explore CTC biology. To our knowledge, our work represents the first use of cell suspension mass cytometry to characterise CTCs.

When discussing CTC positivity and quantification, with our protocol we detected CTCs in almost all (93%) of the HNSCC patients we analysed, with a range of 2–24 CTCs/ml. In comparison to a previous study using microfluidic enrichment in a HNSCC cohort, Kawada et al. detected CTCs in 90% of advanced HNSCC patients with a mean cell count in the region of 20 CTCs/ml [29]. These similar results compared to our findings build confidence in our enrichment and detection strategy. As will be discussed, our data should illustrate that single-CTC resolution proteomic data is required to provide the ability to identify potentially novel CTC sub-groups. Furthermore, we demonstrated how conventional RNAseq ‘bulk CTC analysis’ lacked the sensitivity to identify intra-patient CTC phenotypic heterogeneity, that could prove to be clinically relevant. Aside from the optimisation and validation of a mass cytometry protocol, the key finding from this pilot study was that CTCs predominantly clustered based upon epithelial-EMT status. This was despite considerable intra-patient CTC heterogeneity of functional phenotypic markers. While we accept our findings are from a feasibility study on a pilot cohort; there were, nevertheless, some notable patterns when we applied high-dimensional clustering analysis. We discuss them in turn below, in relation to current evidence, to provide a platform for future validation studies.

While previous reports have mostly grouped CTCs into binary categories of ‘epithelial’ and ‘mesenchymal’ based upon single marker expression profiles [19, 30] our high-plex phenotyping data also detected intermediate mesenchymal transitional states in CTCs. For example, the classically described EMT transcription factors Twist and Snail were variable in our ‘early’ and ‘advanced’ EMT CTC groups, as were the phenotypic markers associated with them (such as proliferation or stemness). Phenotypic EMT plasticity has been well described in tumours [31], being correlated with progression and metastasis [32]. Recently, in a pancreatic cancer cohort, Semaan et al. identified that patients with an increased proportion of ‘partial EMT’ CTCs (i.e. those CTCs expressing both epithelial and mesenchymal markers) demonstrated significantly worse progression-free and overall survival [33]. In addition, they observed that this partial EMT sub-group made up the majority of CTCs identified in their samples, a similar pattern to our data and that observed in other studies [34]. This finding emphasises the need for CTCs to be enriched without reliance on a specific marker in order to provide an unbiased assessment of the entire CTC population. While previous reports have identified EMT CTCs as being prognostic of treatment response in the recurrent/metastatic setting in HNSCC [35], long-term outcome data in larger cohorts is lacking. However, if the acquisition of a mesenchymal CTC phenotype is linked to metastasis and a poor prognosis, then understanding the regulatory EMT regulatory pathways underpinning this transition in CTCs is of paramount importance. The addition of phosphoprotein-specific antibodies to mass cytometry panels has proved a powerful tool to illuminate cellular signalling pathways [36]. Using these antibodies in our CTC analysis, we detected a notable pattern of pCREB upregulation in EMT sub-groups. Phosphorylated CREB is an oncogenic transcription factor regulating cell proliferation and survival [37], and our preliminary data suggests it could act as a driver in EMT CTCs. In addition, pERK was highly expressed in early-EMT sub-groups but not advanced-EMT CTCs. Notably, both CREB and ERK are reported to be potential druggable targets in cancer [38, 39].

With the advent of immune checkpoint therapeutic agents, research across multiple cancer types has sought to characterise the expression of immune regulatory molecules, including in HNSCC [8, 22]. While current evidence is equivocal regarding their utility as a prognostic biomarker in HNSCC [40], it has been hypothesised that CTC checkpoint marker expression may represent a novel therapeutic target [41]. Our pilot data suggests expression of immune checkpoint markers may correlate with CTC EMT sub-group type, with EMT CTCs generally having decreased immune checkpoint marker expression. Notably, in bladder cancer high immune checkpoint expression was associated with EMT marker expression, with further evidence associating this CTC phenotype with poorer clinical outcomes [42]. Whether this is the case also in HNSCC is a priority for future studies of our CTC phenotyping method.

Few studies have sought to correlate tumour and CTC epithelial-EMT phenotype [19, 43]. In previous work, utilising low-plex (flow cytometry) CTC EMT characterisation, we reported no association between tumour and CTC EMT states [19]. Our mass cytometry analysis of CTCs supports this result. Despite using PCA analysis to successfully group patients into epithelial or EMT groups based upon CTC profiles, this grouping did not correlate with tumour EMT gene expression profiles. Similar findings are reported from other studies in HNSCC, for example Chikamatsu et al. compared tumour and CTC PD-L1 expression in a HNSCC cohort (*n* = 23), noting that in the majority of cases it was not concordant (56.5%) [22]. By definition CTCs are metastatic cells originating from a tumour, however increasing evidence points towards heterogeneity between these two. While our cohort is only a small pilot, our data lends further evidence to the concept of EMT being a CTC survival mechanism, which may be independent to tissue expression, whereby CTCs adapt in the circulation to become pro-metastatic cells [44].

Our study has several limitations. Being a feasibility study to develop a new technological approach to CTC analysis, our patient cohort was small and not ethnically diverse. Furthermore, our cohort was predominantly an oral SCC subsite of HPV-negative cancers, skewed towards advanced stage of disease. Therefore, the generalisability of our findings to other HNSCC cohorts is unknown. It was outside the scope of our project to perform single-cell RNAseq of CTCs to generate data against which to validate our mass cytometry phenotyping. Instead, we validated against bulk RNAseq data of crude CTC preparations from our patient cohort.

In summary, we demonstrate proof-of-principle that the combination of microfluidic enrichment and mass cytometry analysis can successfully be used to provide single-CTC proteomic analysis. With this protocol, phenotype and activation status of critical intracellular cell signalling proteins can be measured in CTCs and peripheral blood immune cells, a depth of information beyond that available from conventional bulk CTC gene expression profiling. The heterogeneity of CTC EMT sub-groups and their relationship to target markers, in particular immune-checkpoints, is notable – potentially providing both an avenue for therapeutic targeting, but also a means of therapeutic escape in ‘immune low’ CTCs. We provide a potential platform for future proteomic based single-CTC studies in larger cohorts.

### Supplementary information

Supplementary table 1

## Data Availability

Data is available on request from the corresponding author.
