# Supplementary material for: Feasibility of mass cytometry proteomic characterisation of circulating tumour cells in head and neck squamous cell carcinoma for deep phenotyping
Source: Br J Cancer. 2023 Sep 21;129(10):1590–8. doi: 10.1038/s41416-023-02428-2 (PMC10645808; doi:10.1038/s41416-023-02428-2)
Supplement: Supplementary file 1 — Supplementary table 1 [file 41416_2023_2428_MOESM1_ESM.docx]

| **Metal** | **marker** | **clone** | **Supplier** | **Extracellular/ Intracellular** |
| --- | --- | --- | --- | --- |
| **089 Y** | CD45 | HI30 | F |  |
| **106 Cd** | B2M, CD298 | 2M2, LnH-94 | BL |  |
| **110 Cd** | CD14 | W052 | BL |  |
| **111 Cd** | Vimentin | E5 | SC |  |
| **112 Cd** | CD66b | 6/40c | BL |  |
| **113 Cd** | CD19 | HIB19 | BL |  |
| **114 Cd** | CD44 | IM7 | BL |  |
| **115 In** | CD3 | UCHT1 | BL |  |
| **116 Cd** | CD4 | RPA-T4 | BL |  |
| **141 Pr** | EpCAM | 9C4 | BL |  |
| **143 Nd** | PARP | F21852 | F |  |
| **144 Nd** | CD31 | WM59 | F |  |
| **146 Nd** | CD86 | FUN1 | BD |  |
| **147 Sm** | Twist | Twist2C1a | SC |  |
| **148 Sm** | PD-L2 | 24F.10C12 | BL |  |
| **149 Sm** | CD25 | 2A3 | F |  |
| **150 Nd** | pSTAT5 | 47 | F |  |
| **152 Sm** | pAKT | D9E | F |  |
| **153 Eu** | pSTAT1 | 58D6 | F |  |
| **154 Sm** | E-cadherin | 67A4 | BL |  |
| **155 Gd** | PD1 | EH122H7 | F |  |
| **156 Gd** | p38 | D3F9 | F |  |
| **158 Eu** | pSTAT 3 | 4/P-STAT3 | F |  |
| **159 Tb** | CD133 | S16015F | BL |  |
| **160 Gd** | CD39 | A1 | F |  |
| **161 Dy** | CTLA4 | 14D3 | F |  |
| **163 Dy** | CD56 | NCAM162 | F |  |
| **164 Dy** | pan-keratin | AE1 | SC |  |
| **165 Ho** | PD-L1 | MIH2 | BL |  |
| **166 Er** | CD24 | ML5 | F |  |
| **168 Er** | Ki-67 | B56 | F |  |
| **170 Er** | EGFR | AY13 | F |  |
| **171 Yb** | pERK | D13144E | F |  |
| **172 Yb** | CD163 | GHI61 | BL |  |
| **174 Yb** | Snail 1 | G-7 | SC |  |
| **176 Yb** | pCREB | 87G3 | F |  |
| **194 Pt** | CD73 | AD2 | BL |  |
| **195 Pt** | CD8 | SK1 | BL |  |
| **196 Pt** | HLA-DR | L243 | BL |  |
| **198 Pt** | HLA-ABC | W632 | BL |  |
| **209 Bi** | CD47 | CC2C6 | F |  |

**Supplementary table 1: Mass cytometry panel.** Protein marker listed with metal isotope tag, antibody clone and supplier. BL = Biolegend, F = Fluidigm, SC = Santa Cruz. Cellular location – red = intracellular, purple = extracellular.
